# A Text-Book of the Practice of Medicine

**Published:** 1901-10

**Authors:** 


A TEXT-BOOK OF THE PRACTICE OF MEDICINE. By James M. Anders, M. D., Ph. D., LL. D., Professor of the Practice of Medicine and Clinical Medicine in the Medico-Chirurgical College, Philadelphia, Etc. Illustrated. Fourth edition. Thoroughly Revised. Pages, 1296. Price, $5.50 cloth. Philadelphia: W. B. Saunders & Co. 1900.

Although the fourth edition of Anders' Practice followed closely upon the third, many and important additions have been made. The section on diseases of the digestive system has been very materially improved, and the late advances on this subject have been recorded with practical application.

The author has rearranged part of the book where rearrangement seemed necessary for convenience to the student and practitioner. Special stress is laid upon the modern methods of diagnosis and the advances in therapeutics. Differential diagnosis is particularly emphasized.

This work has rapidly become a standard, and may be found in the libraries of most up to date physicians. T. J. B.

